# Consensus statement on chronic pain treatment in cancer survivors

**DOI:** 10.1007/s00540-024-03427-0

**Published:** 2024-12-04

**Authors:** Keiko Mamiya, Hiroki Iida, Masako Iseki, Shigeki Yamaguch, Hiroshi Yonekura, Hiroshi Ueno, Toshifumi Kosugi, Takeshi Sasara, Yumiko Takao, Toshifumi Takasusuki, Saori Hashiguchi, Naomi Hirakawa, Yoko Sugiyama, Keiko Yamada, Kenji Yamamoto

**Affiliations:** 1 Division of Palliative Medicine, Shinshu Cancer Center, Shinshu University Hospital, 3-1-1 Asahi, Matsumoto, 390-8621 Japan; 2 Gifu University/Anesthesiology and Pain Relief Center, Central Japan International Medical Center, Minokamo, Japan; 3 Department of Anesthesiology and Pain Medicine, Faculty of Medicine, Juntendo University, Bunkyō, Japan; 4 Department of Anesthesiology, Dokkyo Medical University School of Medicine, Mibu, Japan; 5 Department of Anesthesiology and Pain Medicine, Fujita Health University Bantane Hospital, Nagoya, Japan; 6 Department of Anesthesiology, Kyoto Prefectural University of Medicine, Kyoto, Japan; 7 Department of Palliative Care, Saga-Ken Medical Center Koseikan, Saga, Japan; 8 Yuuaikai Tomishiro Central Hospital, Total Pain Center, Tomigusuku, Japan; 9 Department of Pain Medicine, Hyogo Medical University Hospital, Nishinomiya, Japan; 10 Department of Palliative Medicine, St. Marianna University School of Medicine, Kawasaki, Japan; 11 Department of Anesthesiology and Pain Clinic, Hirakawa Hospital, Tokyo, Japan; 12 Department of Palliative Care, Hokkaido Cancer Center, Hokkaido, Japan

**Keywords:** Cancer survivors, Chronic pain, Statement

## Abstract

In September 2023, the Japan Society of Pain Clinicians (JSPC) issued this consensus statement on chronic pain treatment in cancer survivors. With recent advances in the early diagnosis and treatment of cancer, its prognosis has improved, so prolonged pain in cancer survivors is considered to represent chronic pain and should be addressed. In this statement, we emphasize that not all cancer survivor pain is cancer pain. Pain that is not cancer pain should be managed with analgesics other than opioids and nerve blocks, and pain that persists despite this approach should be treated as non-cancer chronic pain so as to prevent opioid overuse. In addition, cancer survivors at any stage of disease have a potentially life-threatening condition and constantly carry the fear of cancer recurrence. Therefore, even non-cancer pain should not be treated in the same way as general chronic pain, but should be managed with consideration of emotional distress. In the future, we plan to create educational tools for healthcare professionals and to conduct online seminars, both with the goal of providing cancer survivors with appropriate assessment and treatment of chronic pain.

## Introduction

In September 2023, the Japan Society of Pain Clinicians (JSPC) issued this consensus statement on chronic pain treatment in cancer survivors. The Working Group (WG) chair was Keiko Mamiya, and the WG members were Hiroki Iida, Masako Iseki, Shigeki Yamaguchi, Hiroshi Yonekura, Hiroshi Ueno, Toshifumi Kosugi, Takeshi Sasara, Yumiko Takao, Toshifumi Takasusuki, Saori Hashiguchi, and Naomi Hirakawa. The collaborators were Yoko Sugiyama, Keiko Yamada, and Kenji Yamamoto.

Cancer survivors are described as “people who are diagnosed with cancer and live with various problems afterwards.” One of the problems faced by cancer survivors is pain. With recent advances in the early diagnosis and treatment of cancer, the prognosis of cancer has improved, so prolonged pain in cancer survivors is considered to be chronic pain and should be addressed. To date, the American Society of Clinical Oncology (ASCO) has published “Clinical Practice Guidelines for Chronic Pain in Adult Cancer Survivors” [1], and the International Classification of Diseases 11^th^ Revision (ICD-11) includes “chronic cancer-related pain” as a specific disease. The background of these efforts was the inappropriate use of opioid analgesics as a result of overconfidence in these agents, and this overuse has become a social problem.

In Japan, however, the improper use of opioid analgesics has not yet become a social problem due to strict regulations of these drugs. However, a report on the current status of opioid use disorder in Japan clearly showed that improper use of opioid analgesics is becoming more common in patients with cancer, and the use of opioid analgesics for cancer pain and post-treatment pain in patients with cancer is a risk factor for subsequent opioid misuse [2]. It was also noted that to prevent misuse and overdose involving opioid analgesics, adherence to universal precautions must be as important for patients with cancer as it is for non-cancer patients. This is particularly the case because healthcare providers lack a sufficient understanding of chronic pain.

## Objective

**Fig. 1:**
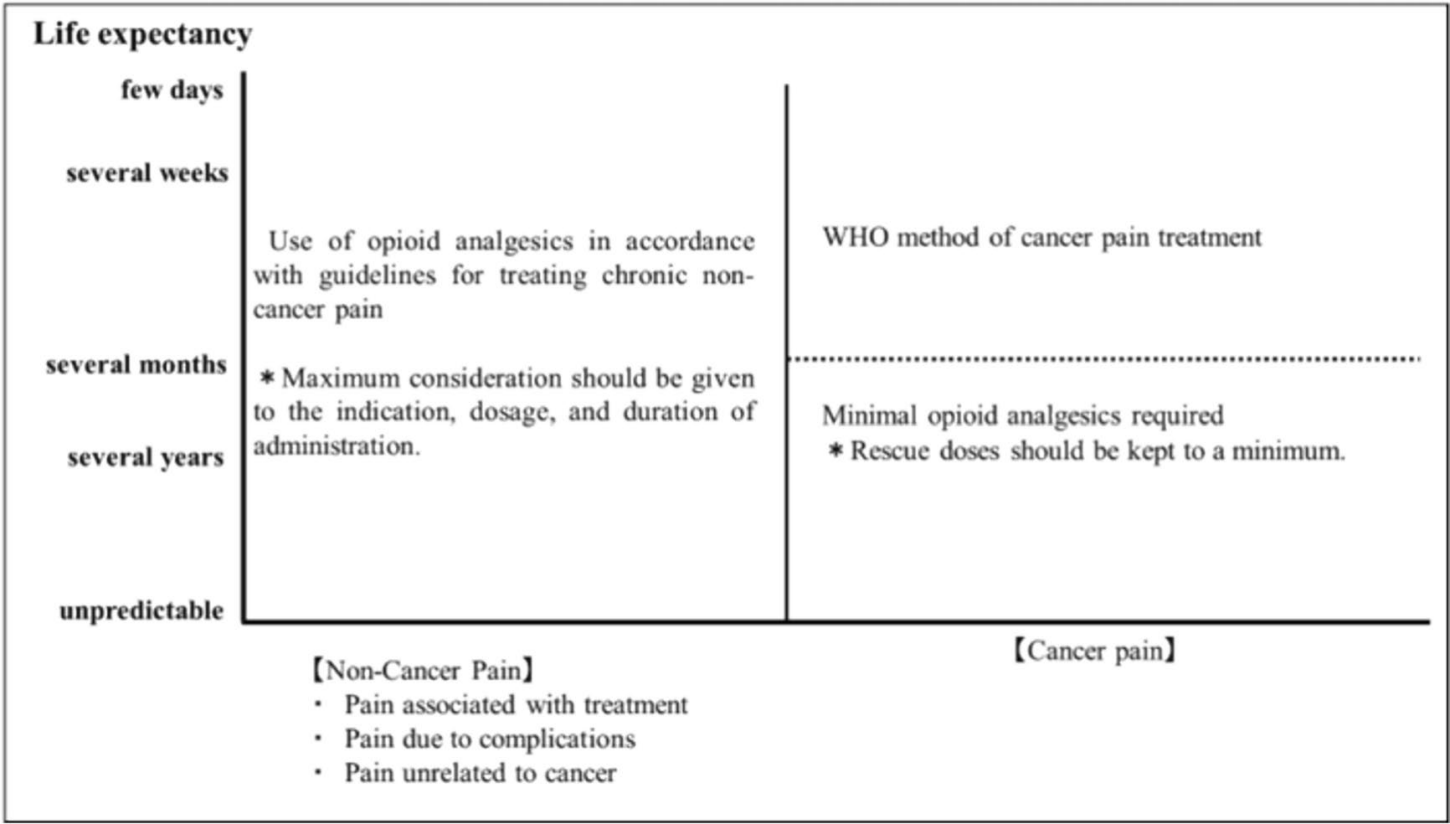
Causes of pain in cancer survivors (adapted from Ref. [3])

The purpose of this statement is to demonstrate the significance of the dissemination of proper chronic pain treatment based on the evidence accumulated to date, with a focus on the proper use of opioid analgesics as shown in Fig. 1 [3]. The statement aims to address the issue of chronic pain among cancer survivors, which will be increasingly prevalent in the future. It is not intended to restrict the use of opioid analgesics.

## Definition of cancer survivor

In developing a statement on the treatment of chronic pain in cancer survivors, we first considered and discussed the definition of a cancer survivor. Although there are various such definitions, due to complicated characteristics of managing chronic pain, this statement defines a cancer survivor as “a person in any stage of life from the time that cancer is diagnosed or suspected to the end of life, rather than only a person whose cancer is cured.” In short, we believe it is more accurate to define a “cancer survivor” as “someone who has been affected by cancer” rather than as a “survivor.”

## Target audience

**Fig. 2:**
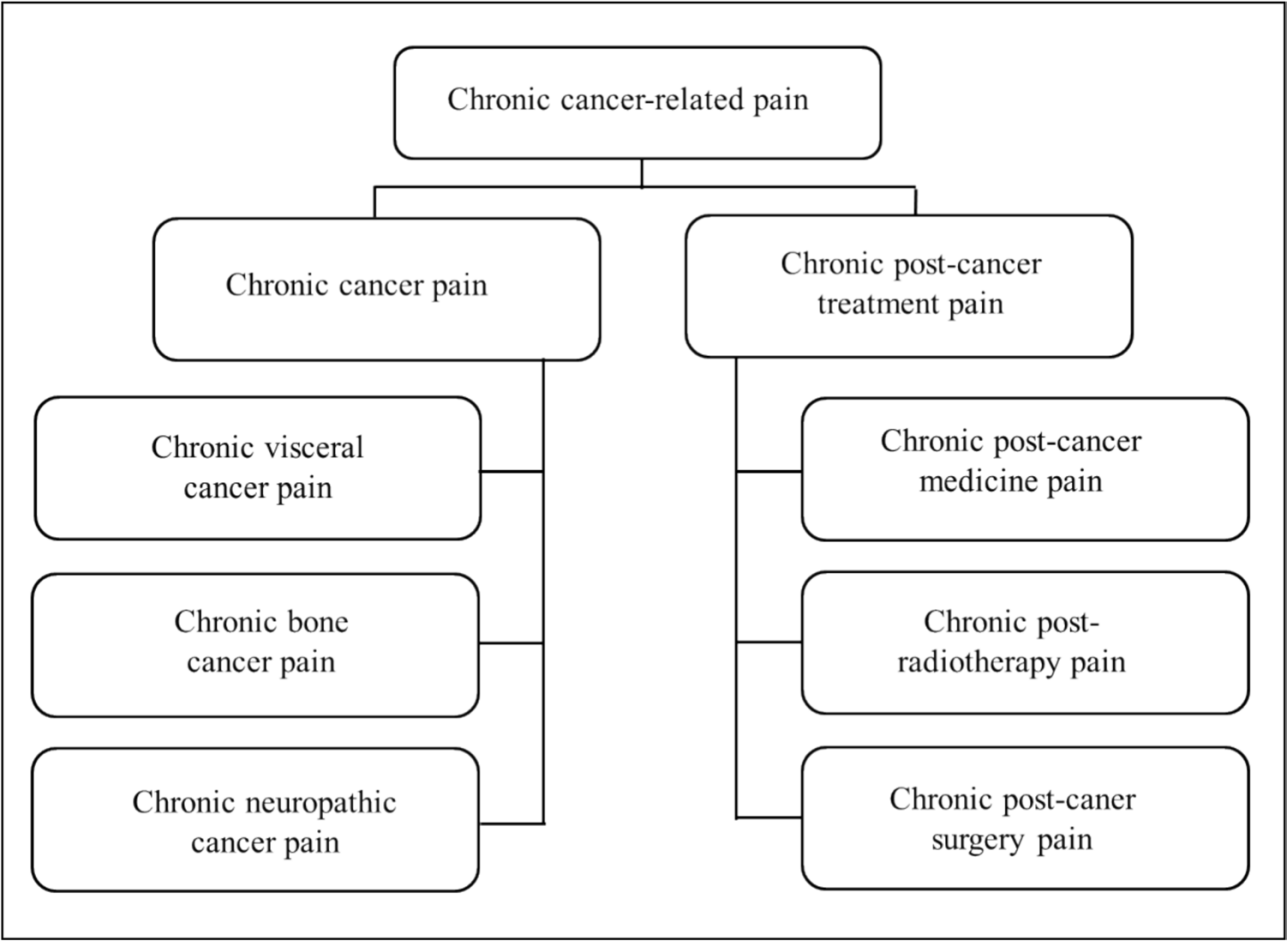
Chronic cancer-related pain (ICD-11) (modified from Ref. [4])

Chronic pain of cancer survivors in this statement includes not only chronic cancer pain (pain directly caused by cancer) and chronic post-cancer treatment pain (pain related to cancer treatment), as described in ICD-11 “Chronic cancer-related pain” [4], but also all pain that occurs before or after cancer diagnosis. In short, chronic pain in cancer survivors includes all pain indicated in the ICD-11 classification of chronic pain (Fig. 2) [4].

Given that the development of cancer treatment and the efforts of healthcare professionals have improved the survival rate and number of cancer survivors, and that cancer is now considered a chronic disease rather than an incurable disease, we would like to emphasize that treatment for cancer survivors who are aware of chronic pain is already a universal part of regular medical care. We hope that this statement will be widely disseminated to all healthcare professionals, not just those who specialize in cancer.

Definition of opioid analgesics: “Opioid analgesics” is a generic term for drugs that exert their analgesic effects by acting on the receptors to which opium binds (opioid receptors).

## Search strategy, levels of evidence, and strengths of recommendation

### Systematic review

A systematic review of the literature on chronic pain treatment in cancer survivors was conducted in MEDLINE (PubMed) in November 2021. Clinical questions (CQs) were defined, and a systematic literature search was conducted for each CQ. Key words were referenced from the 2016 ASCO publication “Management of Chronic Pain in Survivors of Adult Cancers: American Society of Clinical Oncology Clinical Practice Guideline” [1]. Since the concept of “cancer survivor” is new and there is little evidence at this time, we included adult cancer survivors with chronic pain as eligible patients, as well as patients in other adult populations who are at risk of chronic pain. Literature on types of acute pain, such as postoperative pain, was excluded. The time period covered was from January 2014 to November 2021, which was after the publication of the ASCO guidelines. Due to the small number of reports, the study design was not limited to systematic reviews or randomized controlled trials (RCTs) in cancer survivors. After confirming that the pre-defined key articles were included, the literature search formula was finalized, and the date of the literature search in PubMed and the number of hits were recorded. The search formula used in this statement is shown in the Appendix. Hand searching of reference lists and citations of previous articles were conducted additionally as appropriate. Results were limited to studies published in English. Existing practice guidelines or systematic reviews, if available, were selected after assessing the quality, currency, and relevance of the studies.

### Certainty of evidence and strengths of recommendation

Certainty of evidence and the strengths of guideline recommendation were based on the “Minds Manual for Guideline Development 2020 ver. 3.0” [5] and the “GRADE System for Clinical Practice Guideline 3^rd^ Edition” [6].

The overall certainty of evidence for outcomes was specified as follows.

- A (high): We are very confident that the true effect lies close to that of the estimate of the effect.
- B (moderate): We are moderately confident in the effect estimate.
- C (low): Our confidence in the effect estimate is limited.
- D (very low): We have very little confidence in the effect estimate.

Four factors were considered in determining the level of recommendation: overall certainty of evidence, balance between desirable and undesirable effects, values and preferences, and cost and resource use. The strength of recommendation was specified and presented as follows.

1. Strong.
2. Weak.

The certainty of the evidence (A, B, C, or D) and the strength of the recommendation (1 or 2) were combined in each statement. When it was not possible to determine the degree of certainty or strength of recommendation, one or both were not stated.

### Criteria for recommendations and consensus building

A draft version of this document was distributed to each WG member for review and comments. WG members were asked to confirm the text. After feedback was received, a consensus-building meeting was held with all members in attendance. Consensus on each proposed recommendation was reached when at least 80% of members agreed. Discussions and revisions continued until the consensus criteria were met.

## Public comments from related academic societies

The WG solicited public comments on the draft version of this document from members of the Japan Society of Pain Clinicians and related academic societies. The WG discussed the comments received and decided whether to accept or reject them.

## Clinical questions, recommendations, summary statement, and commentary

### Are opioid analgesics effective in the treatment of chronic pain in cancer survivors?

#### Clinical question: what are the optimal prescription parameters (target disease, dosage, duration of administration, response to transient increase in pain, countermeasures against side effects, etc.)?

##### Summary statement

- Opioid analgesics may be used only when non-opioid analgesics are ineffective and opioid analgesics are effective.
- The method of use depends on the prognosis and whether or not the pain is caused by cancer.
- If the prognosis is months or less and the pain is directly caused by cancer, cancer pain management according to WHO Analgesic Guidelines is indicated, and the use of rescue medication is also acceptable.
- In cases of direct cancer pain with a prognosis of months to years, cancer pain management according to WHO Analgesic Guidelines is indicated, but the use of rescue medication should be kept to the minimum necessary.
- For non-cancer pain, opioid analgesics should be prescribed according to the Guidelines for Prescribing Opioid Analgesics for Chronic Non-Cancer Pain only when non-pharmacologic therapies such as non-opioid analgesics and nerve blocks are ineffective, regardless of prognosis. A maximum dosage of oral morphine equivalent of 60 mg/day is recommended, and the dosage should not exceed 90 mg/day. The target duration of treatment should be 3 months and should not exceed 6 months, after which the drug should be withdrawn or the dose reduced and the patient reevaluated.

##### Commentary

With advances in cancer treatment, cancer survivors are being treated for longer periods of time and their prognosis is improving. Cancer survivors are also experiencing pain for a longer period, and it is necessary to correctly assess each survivor’s situation and respond to pain according to each situation. It is also necessary to assess whether the pain is directly caused by cancer or is non-cancer pain (treatment-induced pain, cancer-related pain, or non-cancer-related pain). If the prognosis is months or less and the pain is directly caused by cancer, cancer pain management according to WHO Analgesic Guidelines is indicated, and the use of rescue opioid analgesia is also acceptable. In cases of direct cancer pain with a prognosis of months to years, cancer pain management according to WHO Analgesic Guidelines is indicated, but the use of rescue medication should be kept to the minimum necessary. For pain caused by factors other than cancer, regardless of prognosis, opioid analgesics should be prescribed in accordance with the “Guidelines for Prescribing Opioid Analgesics for Non-Cancer Chronic Pain” only when non-opioid analgesics are ineffective and non-pharmacologic therapies such as nerve blocks are ineffective [7]. Oral morphine equivalents are recommended up to 60 mg/day, with an upper limit of 90 mg/day. The target duration of treatment is 3 months, and should be reevaluated after a maximum of 6 months, with consideration given to drug withdrawal and dose reduction. Prescriptions should be made with attention to the patient’s history of alcoholism and drug dependence, as well as their psychiatric background.

Reported side effects of long-term use of opioid analgesics include constipation, confusion, upper gastrointestinal symptoms (heartburn, nausea, bloating), endocrine disturbances (fatigue, infertility, osteoporosis, decreased libido, menstrual irregularity) due to hypogonadism and increased prolactin secretion, neurotoxicity (myoclonus, worsening of psychological symptoms such as mood swings and memory impairment), risk of exacerbation of opioid-induced pain, and sleep-related disorders (exacerbation of apnea and sleep apnea syndrome due to concomitant use of benzodiazepines) [1]. Immunosuppression and tumor growth have also been reported, although evidence is lacking. For constipation and nausea, laxatives and antiemetics should be prescribed. In addition, to avoid long-term opioid use, it is important to continue pain assessment, aim for dose reduction/discontinuation, administer the lowest dose for the shortest period, and educate cancer survivors and their families [7, 8]. When reducing or discontinuing the dose, gradual reduction is necessary. When pain is under control, dose reductions should be made every 2–4 weeks [7], or by 10% to 25% followed by reevaluation, and temporary dose reductions of 50% to 75% should be made when dangerous side effects such as excessive sedation occur [9].

#### Clinical question: are opioid analgesics effective?

##### Summary statement

- When prescribing opioid analgesics for cancer survivors, determine whether the pain is caused by cancer or non-cancer pain. When prescribing opioid analgesics for chronic pain other than cancer pain, follow the “Guidelines for the Prescribing of Opioid Analgesics for Chronic Non-Cancer Pain.”
- The prevalence of adjustment disorders in cancer survivors is high, and substance abuse associated with long-term opioid analgesics is a concern, as it is in patients with chronic non-cancer pain.
- The risk of substance abuse, gastrointestinal and cardiovascular events, falls and fractures, serious infections, and hospitalization in cancer survivors is high, and these risks increase with higher doses of opioid analgesics.
- When treating chronic pain that requires long-term administration of opioid analgesics, prescription and patient care by a pain management specialist who can conduct rigorous patient screening and monitoring is recommended.

##### Commentary

Opioid analgesics such as morphine, fentanyl, and oxycodone can be prescribed for cancer pain in Japan, and also prescribed for chronic pain in cancer survivors. However, opioid analgesics that can be used for chronic non-cancer pain are limited to morphine tablets, morphine powder, fentanyl patch, and oxycodone (OxyContin® TR tablets). When prescribing long-term opioid analgesics for chronic pain in cancer survivors, it is necessary to consider whether prescriptions should be made in the same way as for chronic pain in non-cancer patients.

A cohort study of 34,188 early stage breast cancer patients in Denmark considered the relationship between opioid analgesics and breast cancer recurrence or development of second cancers [10]. The relationships between the type, duration, and dose of opioid analgesics and the breast cancer recurrence rate were unclear. Similarly, a United States cohort study of 4216 early stage breast cancer patients examined the relationship between long-term opioid analgesics (administered for > 75 days after cancer diagnosis) and breast cancer recurrence or development of a second cancer [11]. There were no significant differences in the rates of breast cancer recurrence or second cancer development between patients who were or were not receiving long-term opioid analgesics.

In Jones et al.’s integrative review of opioid analgesic use among cancer survivors [12], most references defined long-term opioid analgesic treatment as continued use for 3–6 months or longer after radical cancer treatment. In contrast, they defined chronic opioid analgesic treatment as continued use for 6–12 months or longer after the end of cancer treatment. Long-term opioid analgesics are used for 5% to 45% of cancer survivors and are more frequent in patients with head and neck cancer or breast cancer. Most long-term opioid analgesics were administered at dosages of oral morphine milligram equivalents (MME) of less than 20 MME/day. The proportion of patients receiving long-term opioid analgesics at 90 MME/day more than 3 years after completing cancer treatment was higher than in non-oncology patients. Prescriptions for opioid analgesics after completion of cancer treatment were often made by physicians who did not specialize in opioid analgesic treatment. In Canada, family physicians made more than 80% of long-term opioid analgesic prescriptions for patients beyond 5 years after cancer treatment. A history of opioid analgesic use and of chronic pain before cancer diagnosis were shown to be risks for long-term opioid analgesic use in cancer survivors.

Carmona-Bayonas et al., in their review of the literature on long-term opioid analgesic treatment of long-term cancer survivors [13], described the effects of opioid analgesic administration on the human body. Although there are many known effects of opioid analgesics on the immune system, such as decreased immune cell expression, the influences of long-term opioid analgesics on the immune system in long-term cancer survivors are unclear. The prevalence of adjustment disorders (including anxiety and mood disorders) among long-term cancer survivors is 27%–40%, raising concerns that psychological stress may lead to substance abuse during long-term opioid analgesic treatment. In patients with chronic pain, this treatment correlates with the incidence of self-harm, addiction, overdose, and hospital transport. Morphine, fentanyl, and oxycodone increase these risks, but data on long-term cancer survivors are scarce.

A United States cohort study of 38,310 cancer survivors aged 66–90 years who were cured after breast cancer treatment examined the risk of drug-related adverse events [14]. The study showed that opioid analgesics increased the risks of drug abuse, gastrointestinal events, falls, fractures, cardiovascular events, serious infections, and hospitalizations. Compared to patients who received non-opioid analgesics, the risk of drug-related adverse events in patients who received opioid analgesics was 2.3 times greater in the 1–49 MME/day group, and 3.4 times greater in the group receiving more than 50 mg MME/day, suggesting that higher doses of opioid analgesics raised the risk of drug-related adverse events. Although not shown definitively in that study, it can be inferred that most opioid analgesics administered at doses over 50 MME/day were morphine, fentanyl, oxycodone, etc.

As described in the aforementioned studies, long-term and high-dose opioid analgesics are prescribed for chronic pain in cancer survivors, depending on the type of cancer and the curative treatment. In addition, although some adverse events have been identified, the effects of long-term opioid analgesics in cancer survivors are still under study. When prescribing opioid analgesics for chronic pain in such patients, it is necessary to discuss rescue doses, the maximum dose, and the duration of administration from the perspective of adverse event prevention. At present, chronic pain caused by factors other than cancer pain should be treated in the same way as non-cancer chronic pain. Pain management specialists should take measures to prevent and treat opioid-related adverse events and also manage medication adherence.

#### Clinical question: are tramadol, buprenorphine, and codeine effective?

##### Recommendations

- Tramadol is weakly recommended in carefully selected cancer survivors who do not respond to treatment other than opioid analgesics and who have pain-related distress and functional impairment. [2B].

##### Summary statement

- Tramadol is the most prescribed opioid for various types of pain in cancer survivors.
- Tramadol is a second-line medicine for neuropathic pain when adjunctive analgesic drugs fail to provide adequate analgesia.
- Tramadol can be prescribed without a narcotics license because it is classified as an unregulated drug in Japan. Since tramadol is an opioid, patients should be strictly followed, and careless administration and prolongation should be avoided.
- The analgesic effects and side effect frequency of buprenorphine are the same as those of strong opioids such as morphine and oxycodone.
- Insurance coverage for buprenorphine varies depending on the formulation.

##### Commentary

Tramadol is used for various types of pain [15] and is the most frequently prescribed opioid for moderate to severe pain caused by cancer or non-cancer diseases [16, 17]. The metabolite O-desmethyltramadol (M1) has a weak affinity for the μ-opioid receptor and also inhibits the reuptake of serotonin and noradrenaline. Although it is expected to be effective for neuropathic pain, it has a “number needed to treat” of 4.4 and a “number needed to harm” of 4.2 [18], and the “Guidelines for the Pharmacologic Management of Neuropathic Pain” of the Japan Society of Pain Clinicians states that it is the second choice when antidepressants and gabapentinoids (pregabalin, gabapentin, and mirogabalin) do not provide sufficient pain relief [19]. Seizure due to tramadol is a side effect not seen with other opioid analgesics, and caution should be exercised because it can occur not only in the case of overdose but also at the recommended dosage [20]. Although it has been considered that tramadol is not associated with addiction liability even when used for chronic pain over a long period, recent reports have described the elevated, excited, and relaxed effects associated with dependence, as well as inappropriate use due to its lack of regulation as an opioid analgesic [21]. In addition, a cohort study not limited to cancer patients reported increased risks of all-cause mortality, cardiovascular events, and fractures, but there were no significant differences in the risks of constipation, delirium, falls, opioid dependence, or sleep disorders when compared with codeine [22]. In Japan, insurance covers tramadol alone as analgesia for chronic pain and for various types of cancer pain that are difficult to treat with non-opioid analgesics, while the combination of tramadol and acetaminophen is approved for chronic non-cancer pain that is difficult to treat with non-opioid analgesics and for pain after tooth extraction.

Buprenorphine is a partial μ-opioid receptor agonist that has a higher affinity for the μ-opioid receptor than other μ-opioid receptor agonists. Because of its strong analgesic effect, ceiling effect on respiratory depression, and low risk of respiratory depression at analgesic doses, it is the drug of choice for patients with sleep apnea, dependency tendencies, and respiratory disease, as well as those taking benzodiazepines, gabapentinoids, or muscle relaxants [23]. A comparison of extended-release morphine, oxycodone, fentanyl, and buprenorphine pastes showed no difference in analgesic efficacy over the 28-day observation period, as well as no difference in the incidence of adverse effects such as nausea, vomiting, constipation, and drowsiness [24]. Because of its analgesic effect, it is treated as a strong opioid in some countries. The effects of long-term administration on hormone secretion should be considered. Prolactin secretion is enhanced by low doses of buprenorphine (3 to 30 μg/kg) and suppressed by high doses (1,000 to 3,000 μg/kg). A study in opioid-dependent patients reported that patients treated with buprenorphine had preserved testosterone secretion and a lower incidence of hypogonadism than patients on methadone [25]. In Japan, injectable, suppository, and patch formulations are used clinically, but insurance coverage for injectable formulations is limited to postoperative indications, various types of cancer, and myocardial infarction; suppository coverage is limited to postoperative indications and various types of cancer; and patch coverage is limited to chronic back pain and osteoarthritis, which are difficult to treat with non-opioid analgesics.

Since codeine is metabolized to morphine by CYP2D6, its analgesic effect is diminished in patients with low levels of CYP2D6 metabolic activity. After the Centers for Disease Control and Prevention (CDC) issued guidelines for chronic pain [26], codeine prescriptions increased in the United States [27], but there are no studies with high-quality evidence on the use of codeine to treat chronic cancer pain. In Japan, insurance coverage is limited to analgesia regardless of the disease.

### Are medications other than opioid analgesics effective in the treatment of chronic pain in cancer survivors?

#### Clinical question: are non-opioid analgesics (NSAIDs, acetaminophen) effective?

##### Recommendations

- The use of nonsteroidal anti-inflammatory drugs (NSAIDs) and acetaminophen are weakly recommended to relieve chronic cancer pain and improve function in cancer survivors. [2B].

#### Summary statement

- The efficacy of NSAIDs and acetaminophen for neuropathic pain has not been demonstrated.
- NSAIDs and acetaminophen should be used at the lowest effective dose for the shortest possible time, and their efficacy and adverse events should be assessed regularly to determine whether to continue or discontinue their use.

##### Commentary

Non-opioid analgesics are widely used worldwide for mild to moderate pain. NSAIDs have anti-inflammatory and analgesic effects. On the other hand, acetaminophen has analgesic and antipyretic effects but little anti-inflammatory activity in the periphery. These drugs are expected to be effective mainly for nociceptive pain [28]. Although NSAIDs and acetaminophen are frequently used for nociceptive pain in cancer survivors [29, 30], there is little high-quality evidence demonstrating their efficacy. A network meta-analysis of 81 RCTs (10,003 patients) on the treatment of chronic cancer pain found that other than opioid analgesics, codeine-aspirin combination therapy and diclofenac were effective treatments [31]. The 2016 ASCO publication “Management of Chronic Pain in Survivors of Adult Cancers: American Society of Clinical Oncology Clinical Practice Guideline” states that NSAIDs and acetaminophen may be prescribed for the relief of chronic pain and functional improvement if there are no serious drug interactions or contraindications [1]. However, while NSAIDs and acetaminophen may be effective for short-term pain control, their long-term administration and use at high doses should be avoided in light of their adverse effects (see below).

In addition, while neuropathic pain is often a component of chronic pain in cancer survivors, there is no high-quality evidence showing the efficacy of either NSAIDs or acetaminophen for neuropathic pain, and the Japanese “Guidelines for the Pharmacologic Management of Neuropathic Pain” do not recommend their use [19]. The Japanese “Clinical Guide of Management for Chemotherapy-Induced Peripheral Neuropathy” does not provide recommendations for their use due to lack of evidence [32].

Notable adverse effects of NSAIDs include gastrointestinal mucosal disorders, cardiovascular disease, renal disorders, hepatotoxicity, bleeding, and NSAID-exacerbated respiratory disease [33]. The risk of gastrointestinal disorders is higher with high-dose and long-term use of NSAIDs. The risk can be reduced using COX-2 inhibitors or by the concomitant use of proton pump inhibitors [34]. Regarding cardiovascular risk, previous NSAIDs are considered to have the same cardiovascular risk as COX-2 inhibitors, and NSAID use should be avoided in patients with cardiovascular disease [35]. In some countries, their use is contraindicated in patients in the setting of coronary artery bypass graft surgery. With regard to renal impairment, the Kidney Disease: Improving Global Outcomes (KDIGO) guidelines suggest that patients with an estimated glomerular filtration rate (eGFR) < 30 should avoid NSAIDs, and patients with an eGFR < 60 should avoid long-term NSAID use [36]. Other contraindications to the use of NSAIDs include a history of hypersensitivity to NSAIDs, serious blood disorders, serious hepatic disorders, serious cardiac dysfunction, serious hypertension, and late-stage pregnancy. In the treatment of chronic pain in cancer survivors, both the patient’s pain and general condition should be thoroughly evaluated, and if NSAIDs are indicated and effective, the lowest effective dose should be used for the shortest possible duration.

Although acetaminophen is considered better tolerated than NSAIDs, use of more than 2 g/day significantly increases the risk of upper gastrointestinal bleeding (relative risk 3.6 (95% confidence interval [CI], 2.6–5.1)) [37]. Furthermore, in recent years, the efficacy of long-term acetaminophen use for chronic pain in osteoarthritis has been questioned [38]. Therefore, discontinuation of acetaminophen should be considered if regular assessment indicates that it is not providing adequate analgesia. It should be used with caution in patients with hepatic dysfunction.

#### Clinical question: are adjuvant analgesics effective in the treatment of chronic pain in *cancer* survivors?

##### Recommendations

- Gabapentinoids are used for neuropathic pain directly related to cancer in cancer survivors, and are weakly recommended in this context because they can reduce the necessary dose of opioid analgesics. [2B]
- Duloxetine is weakly recommended for chronic pain in cancer survivors because it is more effective for pain due to chemotherapy-induced peripheral neuropathy (CIPN) than other adjuvant analgesics. [2B]
- Corticosteroids should not be used long term in cancer survivors for the sole purpose of relieving chronic pain. [2D]

##### Summary statement

- Adjuvant analgesics such as gabapentinoids and antidepressants, which are first-line agents for neuropathic pain, can also be used for cancer-related neuropathic pain, allowing for a reduction in the dose of opioid analgesics. Gabapentinoids such as pregabalin have been validated for efficacy.
- Pregabalin has been reported to be effective for prolonged pain in postmastectomy pain syndrome and post-thoracotomy pain syndrome, but there are no reports of long-term efficacy.

##### Commentary

Although opioid analgesics are often used for cancer-related neuropathic pain in patients with cancer, gabapentinoids and antidepressants are also used. The use of these adjuvant analgesics is recommended to reduce the dose of opioid analgesics, especially in long-term survivors [39]. The efficacy of pregabalin has been validated [40]. Antidepressants such as amitriptyline and duloxetine may also be effective, but there are few reports on the effects of antidepressants for cancer-related neuropathic pain, and there is no firm evidence [41]. These adjuvant analgesics should be modulated to ensure central nervous system tolerability.

In cancer survivors, quality of life may decrease due to persistent pain caused by cancer treatment, such as prolonged postoperative pain and peripheral neuropathy due to chemotherapy. A study demonstrated the efficacy of pregabalin in patients complaining of postmastectomy chronic pain (PMCP) [42]. There have been several prospective studies on postoperative pain after thoracotomy, many of which have shown the efficacy of pregabalin. However, there are no studies on the long-term effects of pregabalin, and more research is warranted [43]. The use of adjuvant analgesics may allow for the discontinuation or dose reduction of opioid analgesics when the latter are used to treat chronic postoperative pain. However, gabapentinoids should be used with caution in patients with renal dysfunction, and if long-term use is ineffective, discontinuation, dose reduction, or non-pharmacologic treatment such as interventional therapy should be considered. CIPN lasts from several months to several years after chemotherapy, causing sensory disturbance and neuropathic pain that reduce the quality of life of patients with cancer, and 30%–50% of patients develop chronic CIPN. One study showed that duloxetine was effective for CIPN-related pain as well as numbness in the lower extremities [44]. It is also the only drug recommended for CIPN by ASCO [45]. In Japan, the Japanese Association of Supportive Care in Cancer (JASCC) published the “Clinical Guide of Management for Chemotherapy-Induced Peripheral Neuropathy” in 2017 [32]. According to this guide, we are moderately confident in the effect estimate for duloxetine, and the strength of the recommendation is weak. On the other hand, the effectiveness of pregabalin is unclear.

Duloxetine is contraindicated in severe hepatic or renal impairment, and duloxetine administration may increase the risk of suicidal ideation or suicide attempts in patients under 24 years of age. It is sometimes used in combination with opioid analgesics for cancer pain, but it may cause adverse events such as drowsiness and dizziness. Concomitant use with monoamine oxidase inhibitors is contraindicated, and serotonin syndrome may occur with serotonergic agents.

Corticosteroids are recommended by the “WHO Guidelines for the pharmacological and radiotherapeutic management of cancer pain in adults and adolescents,” revised in 2018, to be administered for pain control as an adjunctive agent when indicated [46]. Previous reports provide moderate-quality evidence that corticosteroids may alleviate pain and improve quality of life, and that they may allow for dose reductions of opioid analgesics. Corticosteroids are used for a variety of pain conditions, including pain from bone metastases, but should be prescribed for as short a period as possible. Long-term use in cancer survivors for the sole purpose of relieving chronic pain is not recommended, as there are contraindications in some cases.

#### Clinical question: are Kampo medicine and bisphosphonates effective in the treatment of chronic pain in cancer survivors?

##### Summary statement

- The efficacy of Kampo medicine for CIPN has been widely reported, but no clinical evidence has been established to support its standard use.
- The efficacy of Hangeshashinto for oral mucositis (OM) caused by cancer treatment has been reported in many cases.
- The use of bisphosphonates is recommended for osteoporotic pain in cancer survivors receiving hormone therapy, but caution should be exercised regarding the development of osteonecrosis of the jaw with long-term use.

##### Commentary

Chinese herbal medicine (Kampo medicine) is often effective in relieving pain that is not adequately treated by Western analgesics alone, and the choice of Kampo medicine for cancer pain in cancer survivors may reduce the use of opioid analgesics and improve quality of life.

In cancer survivors, CIPN often continues for a long time, resulting in decreased quality of life. There have been many basic and clinical studies on the treatment of CIPN with Kampo medicine [47]. CIPN is becoming an important issue in terms of maintaining quality of life due to the increasingly long-term survival of cancer survivors. There have been several reports on the efficacy of Goshajinkigan for CIPN [48, 49]. The efficacy of Ninjin’yoeito on CIPN has also been reported [50]. Although there have been several studies on Kampo medicine, clinical evidence supporting the standard use of Kampo medicine for CIPN has not yet been established, and future studies on the mechanisms of neuropathy as well as the efficacy of Kampo medicine are expected. The JASCC guidelines also do not recommend the administration of Goshajinkigan as prophylaxis for CIPN [32]. However, the use of Kampo medicine may reduce the dosage of other drugs. Side effects of Kampo medicine include liver dysfunction and interstitial pneumonia for Goshajinkigan, and pseudoaldosteronism and myopathy for Ninjin’yoeito, and these medicines should be discontinued if they are not effective.

OM occurs in 20% to 40% of patients receiving cancer chemotherapy. The incidence of OM increases from 60 to 85% when blood stem cell transplantation is added, and to 90% when patients receive chemotherapy combined with radiation for head and neck cancer. The effect of Hangeshashinto on OM has been shown to be due to the promotion of oral keratinocyte migration through upregulation of chemokine ligand 12 (CXCL12) via extracellular signal-regulated kinase (ERK) [51]. Studies in patients with colorectal cancer and gastric cancer reported that the mean time to improvement of grade 2 or higher OM was shorter in the group treated with Hangeshashinto than in the placebo group [52, 53]. OM caused by radiation or anticancer drugs is difficult to treat and may require the use of opioid analgesics, but their dosage may be decreased by the concomitant use of Hangeshashinto. Mouth rinses or topical application of Hangeshashinto is associated with fewer side effects than systemic administration.

Patients undergoing hormone therapy, such as patients with breast or prostate cancer, have reduced bone density and are at increased risk of osteoporosis [54]. When bisphosphonates and denosumab are used to treat osteoporosis pain in cancer survivors, they should be used at the indicated dose for osteoporosis [54]. The potential development of osteonecrosis of the jaw should be noted with long-term use of these agents.

### Are non-pharmacologic therapies effective in the treatment of chronic pain in *cancer* survivors?

#### Clinical question: is interventional therapy effective?

##### Recommendations

- Nerve blocks with neurolytics for cancer-related pain in internal organs are effective and may be considered because they can reduce the use of opioid analgesics. [2C]
- Intrathecal analgesia is an effective means of treating intractable cancer pain and may be considered. [2C]

##### Summary statement

- Nerve blocks with neurolytics for visceral cancer-related pain are effective and can reduce the use of opioid analgesics. Early intervention is likely to be effective.
- Intrathecal analgesia is an effective means of treating intractable cancer pain.
- Spinal cord stimulation (SCS) may be considered for intractable cancer pain and pain associated with cancer treatment (e.g., peripheral neuropathic pain caused by anticancer drugs), although the quality of the evidence is low.

##### Commentary

Although it is difficult to conduct a high-quality RCT of interventional treatment for pain in cancer survivors since the number of target patients is small and their prognosis is variable, there is a relatively large number of reports on nerve blocks with neurolytics for cancer-related pain in internal organs. A comparison of two groups of patients, treated with or without a relatively early celiac plexus block (splanchnic nerve block), showed that the group with the block had better pain relief and quality of life [55]. In a report of superior hypogastric plexus block in 180 patients with pain due to pelvic malignancy, pain was reduced by 48% and the use of opioid analgesics was reduced by 55% within the 3 months after the procedure [56]. In an RCT of 50 patients divided into a superior hypogastric plexus block group and an opioid analgesics-only group, pain and the opioid analgesics dosage were reduced by more than 50% in the block group over a 2- to 3-month period [57]. In a study of 14 patients with cancer pain in the perineum who underwent ganglion impar block, 79% of patients experience pain relief and the morphine requirement was significantly reduced 3 months later [58]. In a study of 15 patients with pelvic and perineal cancer pain who underwent superior hypogastric plexus block combined with ganglion impar block with a neurolytic, all patients experienced pain relief and reduced morphine requirements [59].

Overseas, intrathecal analgesia is used for patients with intractable cancer or chronic pain (in Japan, morphine-based intrathecal analgesia for such pain was approved by insurance in December 2020). Abroad, intrathecal analgesia is indicated for patients with cancer pain in whom pain control is difficult with conventional methods or for those who cannot tolerate the side effects of opioid analgesics. Multiple RCTs have reported that intrathecal analgesia has analgesic effects and can reduce the side effects of opioid analgesics [60, 61]. In a study of pain management in patients with cancer pain comparing the use of pharmacotherapy alone with the combination of pharmacotherapy and intrathecal analgesia, the combination treatment resulted in a shorter length of hospitalization and fewer outpatient visits and emergency room visits, as well as reduced medical expenses [62].

A review of SCS for cancer-related pain published in 2020 found that this treatment had only been reported in case reports and a few case series [63]. A report of SCS in 14 patients with chest pain due to lung cancer and another report of SCS in 15 patients with low back pain related to colorectal cancer or anal cancer metastasis both showed that SCS was effective without any complications 1 year later [64]. There have also been many case reports showing that SCS was effective for peripheral neuropathic pain caused by anticancer agents [65, 66].

According to the guidelines of the Polyanalgesic Consensus Conference (PACC), intrathecal analgesia is indicated if pain is difficult to control with conventional pharmacotherapy, there is a fairly good prognosis (at least 3-month survival), pain is localized, and pain is nociceptive or mechanical [67]. Furthermore, SCS is indicated for patients with refractory, neuropathic pain whose disease is relatively stable.

#### Clinical question: are psychotherapy, complementary and alternative therapies, and other therapies effective?

##### Recommendations

- Psychosocial approaches such as psychotherapy, self-management, and psychoeducation are effective in treating chronic pain in cancer survivors.【1B】
- Mind–body therapy for pain in cancer survivors is effective for improving cancer-related pain and may be considered. [2D]
- Cognitive-behavioral therapy (CBT) for cancer survivors should be considered because it can be used alone or in combination to improve pain and resulting psychological states, fatigue, and quality of life. [2C]

##### Summary statement

- Psychosocial approaches such as psychotherapy, self-management, and psychoeducation for cancer survivor pain are effective in improving pain and also physical and mental health well-being.
- Educational interventions involving pain education, self-management education, motivational interviewing, and coaching are effective in improving cancer survivor pain.
- Psychotherapies such as hypnosis, CBT, relaxation, guided imagery, and supportive group therapy, alone or in combination, are effective in improving pain and pain-related psychological states, fatigue, and quality of life.
- Psychotherapies that mainly use mindfulness and meditation, such as mindfulness-based stress reduction (MBSR) and acceptance and commitment therapy (ACT), do not show effects on pain itself, but are effective in improving outcomes of psychological stress such as anxiety, depression, and fatigue.
- Cancer survivors, no matter their disease stage, inevitably face threats to their life and recurrent fears of cancer. Therefore, cancer survivor pain, even non-cancer pain, should not be treated in the same way as general chronic pain, but rather with consideration of these patients’ emotional distress.

##### Commentary

Since cancer survivor pain negatively impacts psychosocial issues such as depression, anxiety, fear of recurrence, fatigue, sleep disturbances, decreased activity, isolation, and low quality of life, assessment of psychosocial status and support are necessary as part of a multifaceted approach to pain [1, 68, 69].

Psychosocial interventions such as educational programs including psychotherapy (hypnosis, cognitive-behavioral therapy, relaxation, guided imagery, mindfulness meditation, and supportive group therapy) have been offered as non-pharmacologic treatment for pain in patients with cancer, either alone or in combination with exercise therapy or complementary and alternative therapies, and have shown to be effective [70–72].

Regarding psychotherapy for pain in cancer survivors, systematic reviews and RCTs have validated the use of hypnosis, imagery therapy, CBT, MBSR, mindfulness-based cognitive therapy, ACT, and others.

Hypnotherapy has shown moderate to strong effect sizes for acute pain and distress reduction, including distress at the time of cancer diagnosis and treatment, pain associated with cancer-related biopsies, postoperative pain, and stomatitis [73–75]. In addition, an RCT on the combined effects of waking hypnosis and CBT for cancer survivors with depression demonstrated large improvements in catastrophic thinking, distress, and pain (effect sizes of 0.65, 0.93, and 0.82, respectively) [76]. Relaxation techniques with imagery therapy have shown significant pain relief in inpatients with cancer pain, outpatients with chronic cancer pain, and patients with early stage breast cancer [1, 77]. CBT is effective in improving self-efficacy, catastrophic thinking, depression, and anxiety, and numerous studies have shown moderate effect size improvements for pain and hardship in patients with breast cancer [70]. MBSR, which primarily involves meditation, is mildly to moderately effective for chronic pain in non-cancer patients; in cancer survivors, it reduced pain but to a nonsignificant extent, but significantly decreased stress, increased quality of life, and improved sleep disturbances and fatigue [71, 75, 78]. Mindfulness is a component of mind–body approaches such as yoga and qigong [75, 79], and recently there have been many reports combining mindfulness and CBT [73, 75, 80]. ACT has been shown to reduce depression, anxiety, and fear of recurrence in cancer survivors [81], and a systematic review found that it improved psychological flexibility and quality of life, while RCTs revealed that it partially prevented postoperative chronic pain in patients with breast cancer [82].

These psychotherapies have not been tested in cancer survivors other than those with breast cancer, and they should be evaluated in other types of cancer [73, 75, 79, 80]. In Japan, the numbers of therapists and facilities that can provide these psychotherapies for pain are currently limited, and the development of a treatment and support system is desirable.

The usefulness of psychosocial approaches such as pain education and self-management has also been examined in systematic reviews and RCTs [83]. Educational interventions such as information provision, health guidance, and pain management education are mainly provided by healthcare professionals and peer supporters, and interventions such as patient-centered consultation education programs and coaching have been shown to improve patient self-efficacy and pain [73, 75, 83, 84]. Self-management programs with supportive group therapy have been shown to be effective in increasing physical activity and communication with support persons, and in improving fatigue, physical functioning, self-efficacy, mental distress, depression, sleep, and physical pain [85, 86]. A bridging study in cancer survivors from a racial minority population with limited health-related resources evaluated the impact of a 4- to 12-week comprehensive diabetes prevention education intervention, including motivation, nutrition education, and exercise training, and found significant improvements in pain along with improved health status [87]. On the other hand, a survey of primary care physicians in the United States found that they had insufficient knowledge and education regarding cancer survivor pain, and concluded that there is a need for guidelines on the treatment of this type of pain and for information on non-pharmacologic treatments [88, 89].

In recent years, multifaceted psychological and educational support for cancer survivors has become possible through various methods involving online and wearable devices [90]. The effectiveness of this type of support is expected to be verified in the future, and its use to be expanded more widely.

In terms of acupuncture, there are reports of its effectiveness [91].

#### Clinical question: are exercise and other therapies effective?

##### Recommendations

- Consider exercise therapy because of its potential to improve health-related quality of life in cancer survivors. [1B]

##### Summary statement

- A few studies have reported the effectiveness of exercise therapy on pain in cancer survivors. However, systematic reviews and meta-analyses are scarce, and RCTs focusing on breast cancer survivors predominate.

##### Commentary

In clinical practice, exercise therapy plays a crucial role in helping cancer survivors self-manage pain, particularly when it is not due to cancer invasion or metastasis. This therapy aims to lessen reliance on opioid analgesics and other medications. Currently, most RCTs focus on chronic pain in breast cancer survivors, leaving a gap in evidence regarding the effectiveness of exercise therapy in managing cancer pain more broadly. It is imperative to evaluate this effectiveness in cancer survivors with a wide variety of cancer types and to develop more beneficial exercise regimes.

Presently, evidence includes an RCT examining the impact of strength training, aerobics, or aquafitness on pain prognosis in breast cancer survivors [92]. After 1 year, both the strength training and aquafitness groups exhibited significant pain improvement, unlike the aerobics group, which showed no notable change after 1 year and even increased pain after 2 years. Another RCT found that in patients undergoing adjuvant chemotherapy after breast cancer surgery, chemotherapy-induced pain exacerbation was significantly alleviated in the hospital exercise group compared to a home-based pedometer exercise group over 12 weeks [92].

For chronic pain treatment in cancer survivors with CIPN, JASCC’s “2017 Clinical Guide of Management for Chemotherapy-Induced Peripheral Neuropathy” (2017 CIPN Guide) offers specific guidelines. It notes that there is limited evidence regarding the impact of exercise on CIPN, but suggests considering exercise therapy during chemotherapy as this may have beneficial effects on CIPN [32]. The guide recommends 150 min of moderate physical activity and 75 min of high-intensity aerobic exercise weekly in adults (18–64 years), plus at least two sessions of moderate- to high-intensity resistance exercise. The same exercise levels are advised for older adults (65 + years), with adjustments based on individual fitness [32].

In addition, the 2017 CIPN Guide indicates that compression and cooling therapies have prophylactic benefits against CIPN caused by taxane-based treatments [32]. Reports also indicate the potential of virtual reality (VR) technology in treating chronic pain in cancer survivors [93]. A meta-analysis showed significant VR intervention effects on cancer-related anxiety, depression, pain, and impaired cognitive function, opening avenues for future VR applications [94].

#### Clinical question: is multidisciplinary treatment effective?

##### Recommendations

- For chronic pain in cancer survivors, consider a multidisciplinary treatment approach based on education of patients and their families. [2C]
- Inclusion of pharmacists in the multidisciplinary cancer treatment team, either directly or in collaboration with other healthcare professionals, may improve pain metrics in patients with cancer, suggesting the benefit of their involvement. [2C]

##### Summary statement

- Although few RCTs have evaluated the effectiveness of multidisciplinary treatment for chronic pain in cancer survivors, its efficacy is well recognized in clinical practice.
- Since pharmacotherapy plays a central role in the management of cancer-related pain, incorporating pharmacists into the multidisciplinary treatment team is especially important.

##### Commentary

A 2017 systematic review evaluated various individually tailored treatment options within a multidisciplinary approach to cancer pain [95]. This review found that treatment plans designed by teams proficient in pharmacotherapy and other therapeutic methods were most effective [95]. While multiple treatment methods are available, the review emphasizes tailoring and adjusting treatment plans to fit the unique needs of each patient. However, it also points out the challenge in conducting effective RCTs due to the diversity of patients with cancer pain, which makes it more complicated to assess the effectiveness of multidisciplinary treatments.

Pharmacotherapy is crucial for managing cancer pain effectively. Collaboration with pharmacists is deemed important in addressing the pain of cancer survivors [95]. Challenges in implementing pharmacotherapy include patients’ inadequate expression of pain, apprehensions about the impact of drugs on disease progression, and particularly fears regarding the use of opioid analgesics [96, 97]. Pharmacists typically contribute through drug verification, patient education, and management of adverse events [98].

A 2009 systematic review reported that the addition of educational interventions (e.g., by pharmacists) for cancer patient pharmacotherapy, as compared with usual care and assessed using a numerical rating scale (NRS), resulted in an average decrease in mean pain intensity of one point and an average decrease in maximum pain intensity of 0.78 points [98]. A 2022 systematic review and meta-analysis of RCTs indicated that pharmacist intervention significantly lowered pain intensity, with a standardized difference of 0.35 [95% CI −0.55 to −0.16]. In addition, a pooled analysis of non-RCTs demonstrated a reduction in adverse events (odds ratio of 0.69 [95% CI 0.61–0.79]) and an improvement in quality of life (standardized difference of 0.80 [95% CI 0.29–1.32]), underscoring the importance of integrating pharmacists into multidisciplinary cancer treatment teams [99].

Nurses also play a pivotal role in multidisciplinary rehabilitation treatment for cancer survivors. They are essential team members in managing a variety of symptoms, including lymphedema and CIPN pain, in patients with moderate to severe conditions. Nurses assist in introducing exercise therapy to patients with mild to moderate diseases, and facilitate the integration of community exercise programs for patients with mild illnesses, especially when their comorbidities render participation in standard community programs challenging or unsafe [100].

### Do risk assessment and mitigation strategies for opioid analgesics facilitate their proper use in cancer survivors with chronic pain?

#### Clinical question: what do the guidelines indicate?

##### Summary statement

- In North America, where the prescription rate of opioid analgesics is extremely high, guidelines for prescribing opioid analgesics for chronic pain have been established and reported to be of some usefulness.
- Recently, with advances in cancer treatment, there have been reports of increased use of opioid analgesics by cancer survivors and for chronic pain. One of the measures taken in the United States has been the Risk Evaluation and Mitigation Strategy (REMS) to address barriers to the use of opioid analgesics.
- Opinions on the usefulness of these guidelines are divided, and further observation and evaluation are needed.

##### Commentary

In North America, where opioid analgesics use disorders are very serious, guidelines for chronic pain [26, 101] have been issued. They all indicate that non- opioid analgesic treatment should be given priority, and the use of opioid analgesics should be kept to a minimum. In addition, opioid use disorder by cancer survivors has also become an issue, and related to this issue, in 2016 ASCO published “Management of Chronic Pain in Survivors of Adult Cancers: American Society of Clinical Oncology Clinical Practice Guideline” [1].

The new 2016 “CDC Guideline for Prescribing Opioids for Chronic Pain–United States, 2016” are intended to improve communication between clinicians and patients regarding the risks and benefits of prescribing opioid analgesics, improve the safety and effectiveness of pain treatment, and reduce the risks associated with long-term opioid analgesic therapy, including inappropriate opioid use, overdose, and death [26]. The guideline consists of 12 recommendations, the first of which is that non-opioid analgesic therapy is the preferred treatment for chronic pain. Since 2016, certain outcomes resulting from this guideline have been reported, such as a decrease in opioid analgesic-related deaths and a decrease in prescriptions, but it has also been pointed out that opioid analgesics may be discontinued even though this does not benefit the patient [102, 103]. The CDC guidelines were revised in 2022, and in addition to the contents of the 2016 guidelines, they state that adverse events such as acute withdrawal symptoms and suicidal thoughts due to rapid reduction or discontinuation of opioid analgesics should be taken into account, and that flexible measures should be taken for each individual patient [104].

In Canada, the National Opioid Use Guideline Group made recommendations for the safe and effective use of opioid analgesics in 2010, most of which supported the prescription of these drugs [105]. In 2017, “Guideline for opioid therapy and chronic noncancer pain,” which consisted of 10 recommendations based on new evidence, indicated that for patients with non-cancer chronic pain, non-opioid analgesics and non-pharmacologic therapies should be prioritized over opioid analgesic prescriptions. Although one study reported that this guideline is useful, it also showed that it is difficult to apply strictly in actual clinical practice [101].

The 2016 ASCO “Management of Chronic Pain in Survivors of Adult Cancers: American Society of Clinical Oncology Clinical Practice Guideline” provides recommendations for the optimal management of chronic pain in adult cancer survivors [1]. Broadly categorized, the recommendations relate to (1) screening and comprehensive assessment of pain; (2) treatment and care; and (3) risk assessment, mitigation strategies, and preventive measures related to the use of opioid analgesics. In particular, with regard to opioid use disorders, the report presents preventive measures, risk stratification, and recommendations for adherence monitoring [1].

In 2012, the Food and Drug Administration (FDA) mandated REMS for manufacturers and distributors of extended-release/long-acting (ER/LA) prescription narcotics. REMS require all healthcare professionals to attend a program based on the FDA blueprint and to conduct voluntary, REMS-aligned continuing education for prescribers. All of these costs are required to be borne by the manufacturer and distributor. In addition, ER/LA manufacturers and distributors are required to develop medication guides to inform patients about the risks associated with ER/LA opioid analgesics and to monitor and report annually on prescriber knowledge and behavior, and also patient access and safety. While there are a number of articles on the evaluation of REMS, including reports of decreased prescriptions of ER/LA opioid analgesics [106] and improved understanding of opioid analgesic prescribing among participants [107] after REMS implementation, some reports state that the effectiveness of REMS must be further evaluated, for instance regarding reduced opioid analgesic use and unclear effects on patient outcomes [108, 109].

#### Clinical question: are tools evaluating opioid analgesic use beneficial?

##### Summary statement

- Although no tools assessing opioid analgesic use exist specifically for cancer survivors, screening tools are utilized in the United States and Europe to predict barriers to the use of opioid analgesics in patients with chronic pain for whom they are already prescribed or about to be prescribed, and validation of the prescription is strongly recommended.
- Opinions are divided on the usefulness and accuracy of these tools, and further validation and improvement are thought to be needed.
- At this stage, further verification of the validity of the Japanese versions of these assessment tools is considered necessary in Japan.
- In Japan, it is thought that the use of these tools in medical care will lead to the prediction and early detection of opioid analgesic use disorder.

##### Commentary

Below are some typical screening tools.

**Table:** 

Assessment tools	Summary	Verification
SOAPP (screener and opioid assessment for patients with pain)	An assessment tool for predicting disability resulting from the use of long-term medical narcotics in patients with chronic pain. Fourteen items are rated on a 5-point scale, with seven or higher indicating high risk	While some literature has reported the usefulness of SOAPP and SOAPP-R [110, 111], others argue that further validation is needed to prove their usefulness, as well as addressing issues such as its lower sensitivity compared to other tools [112, 113]
SOAPP—R (screener and opioid assessment for patients with pain—revised)	A revised version of the SOAPP, with 24 items rated on a 5-point scale, with 18 or higher indicating high risk	
ORT (opioid risk tool)	Assessment tool for patients with chronic pain who are expected to receive long-term treatment. A score of 0–3 indicates low risk, 4–7 intermediate risk, and 8 or higher high risk	The sensitivity, specificity, positive predictive value, and negative predictive value for predicting medical narcotic use disorder in non-cancer patients are all excellent and have been reported to be clinically useful [114]
DAST (drug abuse screening test)	A simple tool for assessing drug and alcohol use disorders; the DAST-10, DAST-20, and DAST-28 are available	
DIRE (diagnosis, intractability, risk, and efficacy) score	A score predicting pain relief and drug compliance in patients with long-term prescriptions for medical narcotics for non-cancer chronic pain. The questionnaire consists of four components: diagnosis, refractoriness, risk, and efficacy, and each question is rated on a 3-point scale, with 7–13 indicating inappropriateness of long-term prescription and 14–21 indicating appropriateness	A retrospective study demonstrated patient drug compliance after initiation of dosing and reported the usefulness of the tool [115]
COMM (current opioid misuse measure)	One of the most used and trusted tools. Seventeen questions, each scored from 0 to 4 points	A score of 9 or higher raises suspicion of a medication use disorder (sensitivity 71%, specificity 71%) [116]. Some reports have indicated a sensitivity of 77% and specificity of 77% for a score of 13 or higher, indicating its usefulness [117]
PMQ (pain medication questionnaire)	Screening tool for use disorder in patients with chronic pain who have already begun opioid therapy, consisting of 26 questions, each scored on a scale of 0–4 points, with 25 or less indicating low risk, 25–30 indicating the need for caution, and 30 or more indicating that opioid analgesics should be discontinued	The sensitivity and specificity of the simplified PMQ were reported to be 74% and 93%, respectively [118]. On the other hand, another report concluded that the sensitivity and specificity were 36% and 78%, respectively, and were similar to those of SOAPP-R and ORT [119]
PDUQ (prescription drug use questionnaire)	The questionnaire consists of 31 questions, with a score of 10 or higher indicating a high risk of use disorder [120]	
PDUQp (patient version of PDUQ)		

#### Clinical question: is obtaining a consent form useful?

##### Recommendations

- Although there is no direct evidence that obtaining written consent reduces the risk of opioid analgesic misuse when used for chronic pain by cancer survivors, prescribing doctors should consider obtaining written consent and treatment plans in light of the risk of improper use. [2D]

##### Summary statement

- Guidelines in various countries recommend obtaining written consent when initiating treatment with opioid analgesics for non-cancer chronic pain.
- The consent form should be a contractual document that includes information about the goals of treatment with opioid analgesics, the risks involved, and the responsibilities and compliance of the healthcare provider and patient, with a clear mutual understanding of the content.
- Obtaining a consent form may reduce the risk of improper use of opioid analgesics, including overdose, misuse, abuse, and diversion.
- Although there is no evidence that obtaining written consent is useful in reducing the risk of opioid analgesic misuse when used for chronic pain by cancer survivors, written consent and treatment plans should be considered given the risk of improper use.

##### Commentary

Guidelines in the United States and other countries around the world recommend obtaining written consent when initiating treatment with opioid analgesics for non-cancer chronic pain [7, 121–123]. The consent form serves two functions: providing informed consent for treatment and confirming that both the physician and the patient understand the treatment goal and plan [124]. A systematic review of studies examining how the risk of improper use of opioid analgesics was impacted by obtaining consent forms and by urine drug testing concluded, albeit on weak grounds, that both contributed to risk reduction [125].

Consent forms for the use of opioid analgesics for chronic non-cancer pain are provided in the American Academy of Pain Medicine (AAPM) guidelines [126] and the Japan Society of Pain Clinicians’ “Guidelines for Prescribing Opioid Analgesics for Chronic Non-Cancer Pain” [7]. The guidelines of the Japan Society of Pain Clinicians state that consent forms should address the 11 points shown in Table 1 [7]; these include the purpose of opioid analgesic treatment, risks of long-term use, and treatment discontinuation.

**Table 1:** Eleven points that should be included in the consent form

1) Decisions regarding initiating an opioid analgesic prescription, adjusting the dose, or discontinuing treatment, etc., will be made by a physician
2) The ultimate objective of opioid therapy is to improve QOL
3) Clarify the objective of opioid therapy
4) Clearly understand the objective of opioid therapy
5) During opioid therapy, the patient will undergo periodic medical examinations as established by a physician
6) The patient cannot be prescribed opioid analgesics by more than one medical facility
7) Various adverse drug reactions occur as a result of long-term prescription of opioid analgesics
8) Prescription of opioid analgesics is not a treatment that can be continued permanently
9) The patient shall never give opioid analgesics to another person
10) Changing the dosage form or method of use is not allowed
11) If the patient has opioid analgesics that are not needed because opioid therapy has been discontinued or the type of opioid analgesic has changed, they should be immediately returned to the physician (medical facility) that prescribed them

Adapted from [7]

Some healthcare providers who actually use consent forms question whether they reduce the risk of opioid analgesic misuse because their content is difficult to understand and they require a reading comprehension level above that of most patients [127]. A survey in the United States also reported that the percentage of primary care physicians who used consent forms when prescribing opioid analgesics for non-cancer chronic pain varied widely, with an average of 48% (9%–84%) [128].

When limited to chronic pain in cancer survivors, no studies have shown whether obtaining written consent for opioid analgesic prescriptions is useful in reducing the risk of improper use, and future studies are required [1]. A survey of 157 United States healthcare providers who managed chronic pain in cancer survivors reported that 85% said they would obtain written consent for the use of opioid analgesics for chronic pain management in cancer survivors [129]. In addition, obtaining written consent for the use of opioid analgesics for cancer pain has not been actively pursued in the past because by nature, cancer is a progressive disease [124]. In recent years, however, the number of long-term cancer survivors has increased with the evolution of cancer treatments, and cancer pain has become more prolonged. Therefore, in light of the risk that long-term use of opioid analgesics may lead to improper use, obtaining written consent should be considered.

#### Clinical question: is education useful?

##### Summary statement

- As in the case of non-cancer chronic pain, educating healthcare providers and patients and families about the treatment of chronic cancer pain in cancer survivors and the proper use of opioid analgesics is important to reduce their overprescription and to deter their improper use.
- When prescribing opioid analgesics for chronic pain in cancer survivors, United States guidelines recommend educating patients and families about the benefits and risks of long-term prescribing, proper drug management, and the risks of using other sedative medications in combination.

##### Commentary

When prescribing opioid analgesics for non-cancer chronic pain, education of the prescribing physician and the patients and their families is important as a primary measure to prevent the misuse of opioid analgesics. Doctors should be educated about treatment options for chronic pain, including non-pharmacologic therapies, as well as about patient risk assessment and proper prescribing, while patients should be educated about both the benefits of opioid analgesics and their potential risks, including death and side effects such as tolerance, dependence, addiction, and immunosuppression. Appropriate education can reduce the overprescription of opioid analgesics, while at the same time providing proper prescriptions to patients who need them, and may deter them from using opioid analgesics for their euphoric effects [122]. A multifaceted self-assessment package designed to encourage Canadian family physicians to comply with opioid analgesic prescription guidelines was reported to significantly improve patient education as well as physician’s knowledge scores [130].

As a secondary prevention measure in the United States, the CDC has implemented prescription drug monitoring programs (PDMPs) to regularly monitor prescriptions and educate prescribers after they have initiated treatment with opioid analgesics. While some reports have indicated that this program has significantly reduced the use of illicit drugs and the frequency of opioid analgesic misuse [131, 132], others have found that it has not led to a reduction in mortality from opioid analgesic overdose [133], and therefore, the effectiveness of the program is controversial.

In Japan, several opioid analgesics are covered by insurance for non-cancer pain. To ensure the safety of OxyContin® TR tablets, Norspan® Tape, and Fentos® Tape, pharmaceutical manufacturers seek to limit the number of prescribing physicians by requiring prescribers to take e-learning courses. The effects of these courses on safety have not yet been verified.

Education is also important regarding the proper use of opioid analgesics for chronic pain in cancer survivors, and the 2016 ASCO “Management of Chronic Pain in Survivors of Adult Cancers: American Society of Clinical Oncology Clinical Practice Guideline” recommends that prescribers educate patients and families about the risks and benefits of long-term treatment with opioid analgesics, safe drug management, and precautions when opioid analgesics are used with alcohol or other sedative medications [1]. Approximately, 60% to 70% of Canadian primary care physicians who treat chronic pain in cancer survivors believe that guidelines for chronic pain and knowledge about drug and non-pharmacological therapies would be useful, and many of them strongly desire education on treatment options and practical guidelines for chronic pain [89].

## Conclusion

In this document, we introduce the Chronic Pain Treatment Statement for Cancer Survivors, published last year by the Japan Society of Pain Clinicians (JSPC). In this Statement, we sought to emphasize that not all cancer survivor pain is cancer pain. Pain other than cancer pain should be managed with analgesics other than opioids and nerve blocks, and pain that persists despite this approach should be treated as non-cancer chronic pain.

In addition, cancer survivors at any stage of disease have a potentially life-threatening condition and constantly carry the fear of cancer recurrence. Therefore, even non-cancer pain should not be treated in the same way as general chronic pain, but should be managed with consideration of emotional distress.

In the future, we plan to create educational tools for healthcare professionals and to conduct online seminars, both with the goal of providing appropriate assessment and treatment of chronic pain to cancer survivors.

## Data Availability

The data in the present statement are available from the corresponding author upon reasonable request.
