# Correction: Consensus statement on chronic pain treatment in cancer survivors

**DOI:** 10.1007/s00540-024-03441-2

**Published:** 2024-12-18

**Authors:** Keiko Mamiya, Hiroki Iida, Masako Iseki, Shigeki Yamaguch, Hiroshi Yonekura, Hiroshi Ueno, Toshifumi Kosugi, Takeshi Sasara, Yumiko Takao, Toshifumi Takasusuki, Saori Hashiguchi, Naomi Hirakawa, Yoko Sugiyama, Keiko Yamada, Kenji Yamamoto

**Affiliations:** 1 Division of Palliative Medicine, Shinshu Cancer Center, Shinshu University Hospital, 3-1-1 Asahi, Matsumoto, 390-8621 Japan; 2 Gifu University/Anesthesiology and Pain Relief Center, Central Japan International Medical Center, Minokamo, Japan; 3 Department of Anesthesiology and Pain Medicine, Faculty of Medicine, Juntendo University, Bunkyō, Japan; 4 Department of Anesthesiology, Dokkyo Medical University School of Medicine, Mibu, Japan; 5 Department of Anesthesiology and Pain Medicine, Fujita Health University Bantane Hospital, Nagoya, Japan; 6 Department of Anesthesiology, Kyoto Prefectural University of Medicine, Kyoto, Japan; 7 Department of Palliative Care, Saga-Ken Medical Center Koseikan, Saga, Japan; 8 Yuuaikai Tomishiro Central Hospital, Total Pain Center, Tomigusuku, Japan; 9 Department of Pain Medicine, Hyogo Medical University Hospital, Nishinomiya, Japan; 10 Department of Palliative Medicine, St. Marianna University School of Medicine, Kawasaki, Japan; 11 Department of Anesthesiology and Pain Clinic, Hirakawa Hospital, Tokyo, Japan; 12 Department of Palliative Care, Hokkaido Cancer Center, Hokkaido, Japan

**Correction: Journal of Anesthesia** 10.1007/s00540-024-03427-0

In the first sentence of the abstract, ‘the Japanese Society for Palliative Medicine (JSPM)’ should have been changed to ‘the Japan Society of Pain Clinicians (JSPC)’.

In the first sentence of the introduction, ‘the Japanese Society for Palliative Medicine (JSPM)’ should have been changed to ‘the Japan Society of Pain Clinicians (JSPC)’.

In the first sentence of the conclusion, ‘the Japanese Association of Pain Clinics (JAPC)’ should have been changed to ‘the Japan Society of Pain Clinicians (JSPC)’.

The original article has been corrected.

